# Detecting change in stochastic sound sequences

**DOI:** 10.1371/journal.pcbi.1006162

**Published:** 2018-05-29

**Authors:** Benjamin Skerritt-Davis, Mounya Elhilali

**Affiliations:** Electrical & Computer Engineering, Johns Hopkins University, Baltimore, Maryland, United States of America; Technische Universitat Chemnitz, GERMANY

## Abstract

Our ability to parse our acoustic environment relies on the brain’s capacity to extract statistical regularities from surrounding sounds. Previous work in regularity extraction has predominantly focused on the brain’s sensitivity to predictable patterns in sound sequences. However, natural sound environments are rarely completely predictable, often containing some level of randomness, yet the brain is able to effectively interpret its surroundings by extracting useful information from stochastic sounds. It has been previously shown that the brain is sensitive to the marginal lower-order statistics of sound sequences (i.e., mean and variance). In this work, we investigate the brain’s sensitivity to higher-order statistics describing temporal dependencies between sound events through a series of change detection experiments, where listeners are asked to detect changes in randomness in the pitch of tone sequences. Behavioral data indicate listeners collect statistical estimates to process incoming sounds, and a perceptual model based on Bayesian inference shows a capacity in the brain to track higher-order statistics. Further analysis of individual subjects’ behavior indicates an important role of perceptual constraints in listeners’ ability to track these sensory statistics with high fidelity. In addition, the inference model facilitates analysis of neural electroencephalography (EEG) responses, anchoring the analysis relative to the statistics of each stochastic stimulus. This reveals both a deviance response and a change-related disruption in phase of the stimulus-locked response that follow the higher-order statistics. These results shed light on the brain’s ability to process stochastic sound sequences.

## Author summary

To understand our auditory surroundings, the brain extracts invariant representations from sounds over time that are robust to the randomness inherent in real-world sound sources, while staying flexible to adapt to a dynamic environment. The computational mechanisms used to achieve this in auditory perception are not well understood. Typically, this ability is investigated using predictable patterns in a sequence of sounds, asking: “How does the brain detect the pattern embedded in this sequence?”, which does not generalize well to natural listening. Here, we examine processing of stochastic sounds that contain uncertainty in their interpretation, asking: “How does the brain detect the statistical structure instantiated by this sequence?”. We present human experimental evidence employing a perceptual model for predictive processing to show that the brain collects higher-order statistics about the temporal dependencies between sounds. In addition, the model reveals correlates between task performance and individual differences in perception, as well as deviance effects in the neural response that would be otherwise hidden with conventional, stimulus-driven analyses. This model guides our interpretation of both behavioral and neural responses in the presence of stimulus uncertainty, allowing for the study of perception of more natural stimuli in the laboratory.

## Introduction

To understand soundscapes, the brain parses incoming sounds into distinct sources and tracks these sources through time. This process relies on the brain’s ability to sequentially collect information from sounds as they evolve over time, building representations of the underlying sources that are invariant to the randomness present in real-world sounds, while being flexible to adapt to changes in the acoustic scene. Extracting these representations from ongoing sounds is automatic and effortless for the average listener, but the underlying computations in the brain are largely unknown. To better understand how the brain processes real-world sounds, we investigate how the brain builds invariant representations from sounds containing randomness.

Invariant representations of sound sources are referred to in the literature as regularities, where regularity extraction is the brain’s ability to access these representations for use in auditory scene analysis [1, 2]. We differentiate between two types of regularities: *deterministic regularities* that describe a repeating or predictable pattern, and *stochastic regularities* that contain some randomness and are not fully predictable. Deterministic regularities can be as simple as a repeating tone or sequence, or they can be quite complex, for example: two interleaved deterministic patterns [3], an abstract pattern within a single acoustic feature (“falling pitch within tone-pairs” [4]) or one spanning multiple features (“the higher the pitch, the louder the intensity” [5]). The signature trait of deterministic regularities is the absence of ambiguity: a new sound can immediately be interpreted as a continuation of or a deviation from the regularity *with certainty*.

Stochastic regularities, on the other hand, are characterized by the lack of certainty, as their inherent randomness leaves room for multiple possible interpretations of a sequence of sounds. A new sound belongs to a stochastic regularity *probabilistically* according to how well it fits relative to other possible interpretations. For example, consider a sequence of tones with frequencies drawn from an arbitrary distribution, such as in [6]. Each tone could be drawn from the same distribution as the preceding tones or it could be drawn from a new distribution. Given a new tone, deciding between these two alternatives (i.e., “same or different?”) cannot be done with certainty, but rather proportionally to how likely the new tone is given its preceding context. Implicit in this example is that the brain is able to extract meaningful contextual information from previously heard sounds to characterize the stochastic regularity, and represent this abstracted information for interpreting new sounds.

One possible mechanism for how the brain represents stochastic regularities is through statistical estimates, which entails extracting representative parameters from observed sensory cues [7]. The nature and extent of statistics collected by the brain remains unknown. Previous studies have focused on the marginal statistics of tones within a sequence, showing that the brain is sensitive to changes in mean and variance [8, 9]. We refer to these as *lower-order statistics*, describing sounds independent of their context. In the present work, we investigate whether the brain collects *higher-order statistics* about the dependencies between sounds over time; namely, we examine how the brain gathers information about the temporal covariance structure in a stochastic sequence of sounds. We use melody stimuli with pitches based on random fractals, which exhibit long-range dependencies and cannot be described solely by lower-order statistics. We specifically use random fractals because of their ecological relevance: previous work has demonstrated the presence of random fractals in music [10], speech [11], and natural sounds [12] and shown the brain is sensitive to the amount of randomness, or entropy, in random fractal melodies [13, 14].

Change detection experiments are well-suited for investigating regularity extraction, where the task is to detect deviation from an established regularity in a sequence of sounds. A detection can be reported behaviorally or recorded in the brain’s response (e.g., the mismatch negativity, MMN). A correct detection indicates the brain is sensitive to the tested regularity, for a change response is necessarily preceded by knowledge of what is being changed. Change detection experiments in the auditory domain using electroencephalography (EEG) and magnetoencephalography (MEG) have shown the brain is sensitive to a wide range of deterministic regularities [15–17]. Stochastic regularities, however, have mostly been studied using discrimination experiments, where the task is to differentiate between different regularities, with both behavioral [12] and brain imaging results [8, 13, 14, 18, 19] showing the brain is sensitive to various stochastic regularities. Compared to discrimination, the change detection paradigm more closely mirrors how the brain processes sounds in the real world, where boundaries between sound sources are not known *a priori*, but must be inferred from changes in ongoing sound.

The mechanisms needed for change detection may differ depending on the type of regularity. With deterministic regularities, the brain can explicitly test whether each incoming sound deviates from the extracted pattern or not with near certainty. Deviation from a stochastic regularity, on the other hand, emerges gradually as evidence is accumulated over time, causing a delay in the perceived moment of change proportional to the amount of evidence needed to detect the change. This uncertainty unavoidably introduces variability in perception across trials and across subjects, which is particularly problematic for time-locked analyses such as in EEG, where low SNR necessitates many repetitions and precise temporal alignment across trials and subjects to get meaningful results. To account for this variability and facilitate the study of stochastic regularities in change detection, we need a suitable perceptual model of the mechanisms for extracting and using regularities in a changing scene to guide our analysis.

While there have been several theoretical accounts of regularity extraction in the brain [2, 20–23], there are very few mathematical implementations of these concepts into concrete models for tracking regularities in sound inputs. One popular model is the CHAINS model, which examines pattern discovery and competition between alternate partitions of a sequence into concurrent, interleaved patterns [24]. This model has been very insightful in shedding light on principles of bistable perception in stream segregation; yet, its limitation to deterministic patterns impedes its applicability to stochastic regularities in the signal. Another model, IDyOM, initially formulated for application to music perception, uses information-theoretic principles to model auditory expectation, collecting occurrences of previously seen events to build predictions, similar to the n-grams used in language models for speech recognition or text processing [25]. While the IDyOM model is able to capture the statistical structure of both stochastic and deterministic regularities, it is formulated to operate only on a discrete, unordered, small set of possible events, and therefore does not generalize well to sounds that vary on a continuum like pitch or loudness.

In this work, we employ a Bayesian framework to model the tracking of sensory statistics by the auditory system [26]. One of the advantages of Bayesian theory is that it is agnostic to priors and underlying distributions, optimally integrating priors and sensory evidence in the inference process. In particular, this framework makes minimal assumptions on the stationarity of the observed sequence and offers an ideal scheme for tracking statistics and detecting change in underlying probability distributions. Bayesian frameworks have been widely used in various incarnations to model data ranging from financial markets to human behavior in reading-inference, change detection, and reinforcement learning tasks [26–31]. In the present application, this mathematical platform allows us to directly probe the degree of optimality in brain processes observed and test alternative hypotheses for the computations involved.

Here, we adapt this Bayesian framework for perceptual processing to investigate the extent to which auditory statistical information is represented in memory. We introduce perceptual parameters to the model that represent resource limitations (i.e., finite working memory and observation noise) and provide constraints on performance that are valuable to interpret sub-optimal detection performance and variability across listener behaviors. By fitting the model to human behavior from a series of change detection experiments, we can explore questions regarding auditory stochastic regularity extraction: Which statistics are sufficient to explain human behavior? How do the perceptual parameters of the model account for differences in behavior across subjects? Finally, we use the model to guide analysis of EEG data, revealing effects that would be otherwise hidden using conventional EEG analyses.

Results are presented in three parts: the first section presents psychophysics results from a series of change detection experiments, the second section introduces the model and presents results from fitting the model to human behavior, and the third section presents neural results obtained by using the model to guide EEG analysis. We believe this model opens up new avenues into investigating how the brain collects information from stochastic sounds that are more relevant to everyday perception.

## Results

### Psychophysics

**Fig 1:**
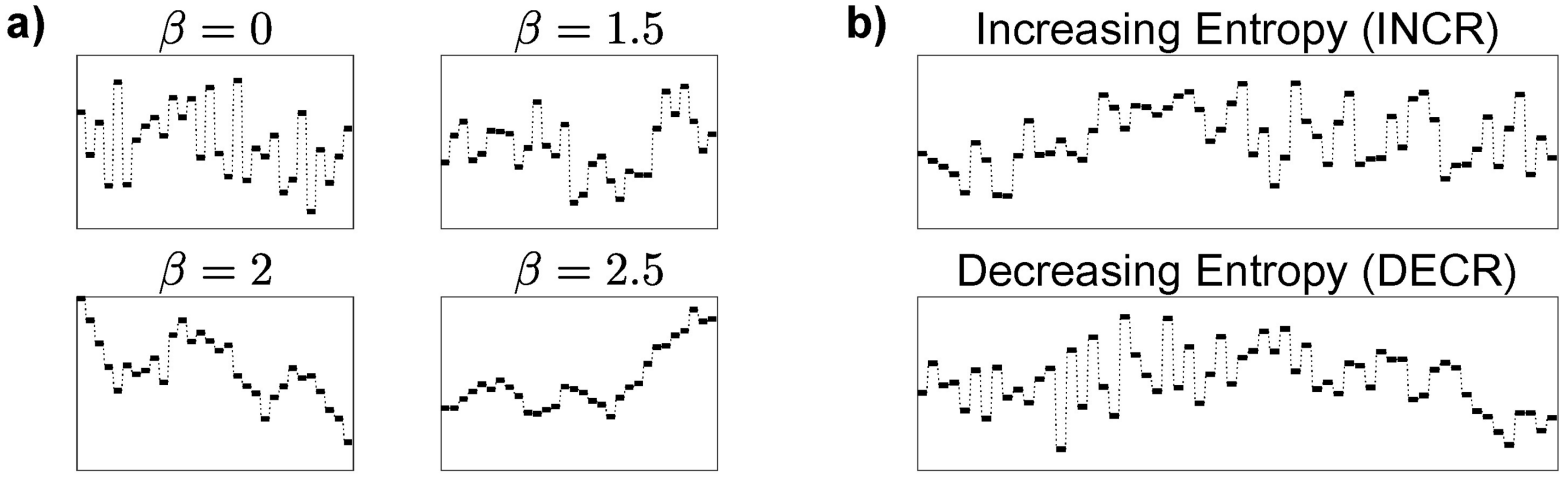
Examples of random fractal melodies. Schematic spectrograms shown with frequency and time along the vertical and horizontal axes, respectively (see S1–S6 Audio. for accompanying audio). a) Melodies at four levels of entropy, parameterized by *β*. Higher *β* corresponds with lower entropy, and vice versa. b) Change stimuli for each change direction; INCR and DECR stimuli always end and begin, respectively, with the highest level of entropy (*β* = 0 or white noise).

A series of experiments probed listener’s ability to detect changes in fractal melodies. Stimuli were constructed from melodies at four levels of *randomness* or *entropy* in pitch (both terms used interchangeably). Melody entropy is parameterized by *β*, where *β* = 0 corresponds to the highest entropy (white noise), and entropy decreases as *β* increases (see Fig 1a for examples of fractal melodies at different levels of *β*). Lower-order statistics (mean and variance) were normalized across the melody. Half-way through the melody, only the higher-order statistics change (see Fig 1b for examples of change stimuli). The task in all experiments was the same: detect a change in entropy of the melody.

#### Experiment 1

We tested how well listeners could detect changes in the entropy of tone sequences and whether the direction of change affected detection performance; see Fig 1b for example stimuli. Listeners (*N* = 10) heard stimuli with three degrees of change in entropy (between *β* = 0 and *β* = 1.5, 2, 2.5) in both directions (INCR and DECR), with control stimuli containing no change (with *β* = 0, 1.5, 2, 2.5). Each melody trial contained 60 tones presented isochronously over 10.5 seconds (175 ms inter-onset interval); there were 150 trials in total, with 15 trials per condition. After each melody trial, listeners responded whether they heard a change and received immediate feedback.

**Fig 2:**
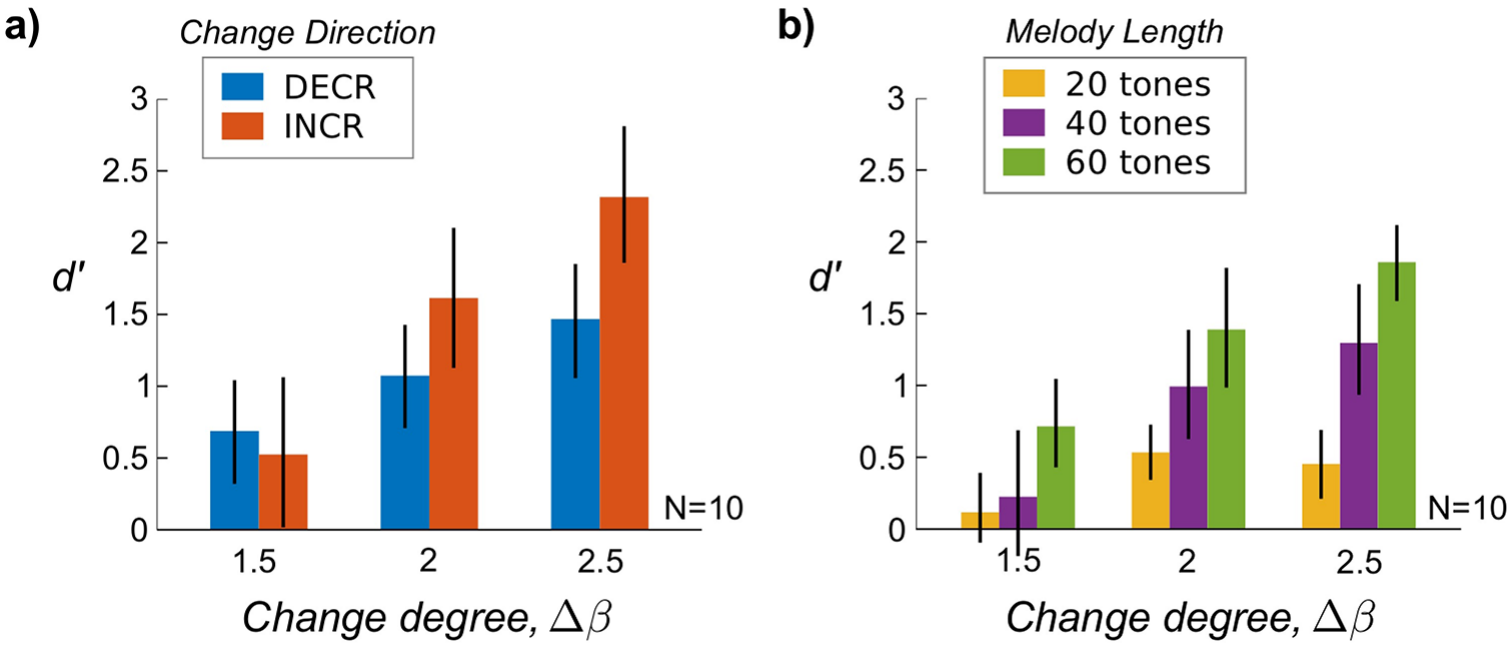
Psychophysics results from Experiments 1 and 2. Average change detection performance (*d*′) across subjects is shown by stimulus condition. Error bars indicate 95% bootstrap confidence interval across subjects. a) In Experiment 1 (*N* = 10), melody entropy changed with different degrees (Δ*β*, abscissa) and in both INCR and DECR direction (color). Detection performance increased with Δ*β* but did not differ by direction, although there was a weak interaction between Δ*β* and direction due to FAs only (see S1 Fig). b) In Experiment 2 (*N* = 10), an additional factor of melody length was introduced (color). Detection performance increased with both Δ*β* and melody length.

Detection performance as measured by *d*′ is shown in Fig 2a; *d*′ comprises both hits and false-alarms (FAs), with higher *d*′ corresponding to better detection performance and *d*′ = 0 corresponding to chance performance. Repeated-measures ANOVAs were used in all analyses to account for between-subject variability. An ANOVA with 2 within-subjects factors (3 change degree x 2 direction) showed a strong effect of degree (*F*(2, 18) = 31.5, *p* < 0.0001), no significant effect of direction, and a significant interaction (*F*(2, 18) = 9.4, *p* < 0.01). We investigated this interaction further by applying ANOVAs separately to hit- and FA-rates (see S1 Fig). The hit-ANOVA showed a strong effect of degree (*F*(2, 18) = 21.9, *p* < 0.0001) but no effect of direction *or* interaction, while the FA-ANOVA showed an effect of entropy level (*F*(3, 27) = 4.7, *p* < 0.01), with FAs increasing with entropy (Note the increase in degrees-of-freedom is due to the 4 levels of *β* for control stimuli). The significant interaction between degree and direction seen in *d*′ above is therefore only due to the effect of entropy on FAs: all DECR stimuli begin with the same high level of entropy (*β* = 0), thus increasing FAs and decreasing *d*′ for DECR compared to INCR stimuli.

It is surprising that there is no effect of change direction on hit-rates. If listeners are relying solely on lower-order statistics, INCR changes should be easier to detect than DECR changes by listening for outliers. We look closely at this effect in a follow-up experiment (Experiment 1b) to contrast response time (RT) to INCR versus DECR changes.

#### Experiment 1b

In this experiment, listeners (*N* = 21) responded *as soon as* they heard a change during melody presentation; otherwise, the stimuli and procedure were the same as in Experiment 1. To confirm that the difference in task itself had no effect on detection performance, two-sample t-tests of *d*′ for each condition showed no difference across the two experiments (*p* > 0.05 for all tests, using Bonferroni correction for multiple comparisons). In addition, ANOVAs applied to hit- and FA-rates as in Experiment 1 showed the same significant effects.

A repeated-measures ANOVA applied to the RT data averaged within conditions for change-trials (3 change degree x 2 direction) showed a significant main effect of change degree (*F*(2, 40) = 14.3, *p* < 0.0001) but no main effect of direction and no significant interaction. This confirms the result from Experiment 1, with no effect of change direction on detection performance.

#### Experiment 2

Next, we tested the effect of sequence length on change detection performance. In addition to the same change degree and direction manipulations from Experiment 1, listeners (*N* = 10) heard melodies with different lengths (20, 40, and 60 tones), with the change always occurring at the midpoint of the melody. As there was no effect of change direction on performance seen in Experiments 1 and 1b, we pooled results across INCR and DECR trials. As in Experiment 1, listeners responded whether they heard a change after the melody presentation and received immediate feedback.

Detection performance as measured by *d*′ is shown in Fig 2b. A repeated-measures ANOVA with 2 factors (3 change degree and 3 melody length) showed significant main effects of both change degree (*F*(2, 18) = 23.9, *p* < 0.0001) and melody length (*F*(2, 18) = 17.7, *p* < 0.0001), with a weak interaction (*F*(4, 36) = 2.8, *p* < 0.05). Post-hoc tests indicated the weak interaction was due to chance performance in the most difficult conditions: Δ*β* = 1.5 with lengths of 20 and 40 tones. In separate ANOVAs for hit- and FA-rates (see S2 Fig), hit-rates showed both main effects of change degree (*F*(2, 18) = 10.2, *p* < 0.01) and length (*F*(2, 18) = 29.6, *p* < 0.0001) with no significant interaction, while the FA-rates only showed a significant effect of entropy level (*F*(2, 18) = 14.6, *p* < 0.001) and no effect of length or interaction.

### Model

**Fig 3:**
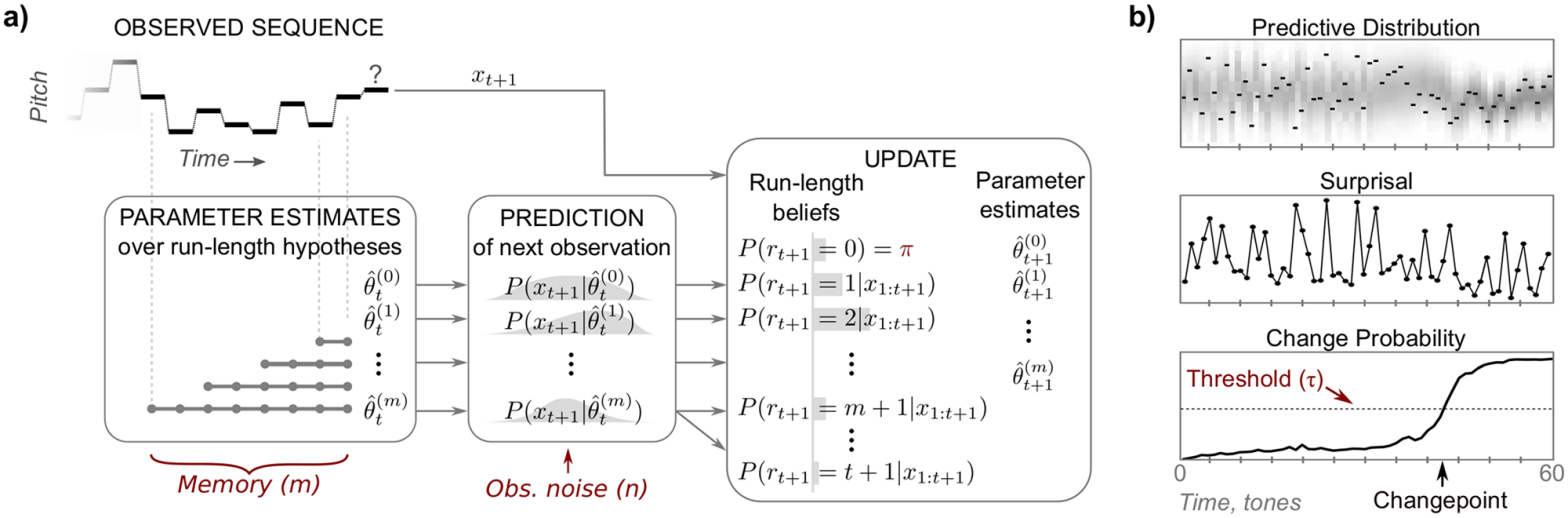
Schematic of perceptual model and model outputs. a) At time *t*, the model contains multiple parameter estimates, ${\hat{\theta }}_{t}^{\left(.\right)}$, collected over run-lengths from 0 up to the memory constraint *m*. Each estimate yields a prediction for the next observation, with increased uncertainty due to observation noise *n*. Upon observing *x*_*t*+1_, the model updates the run-length beliefs using the predictive probability for each hypothesis. Note that the prediction for length *m* is used to update all beliefs with length greater than or equal to *m*, thus limiting the number of past observations used in the update. A new belief with length 0 is added with probability *π*, the change-prior. Finally, parameter estimates are updated with *x*_*t*+1_; these are in turn used to predict the next observation. b) Outputs from the model for an example change stimulus (top, foreground). At each time, the predictive distribution (top, background) combines predictions across run-length hypotheses weighted by their beliefs, thus “integrating out” run-length. Surprisal (middle) measures how well each new observation fits the prediction. The change probability (bottom) is the probability at least one changepoint has occurred, as inferred using the run-length beliefs. The model detects a change if the change probability exceeds the threshold *τ*. Model parameters (*m*, *n*, *π*, *τ*) are in red.

To model brain processes involved in extracting information from stochastic sequences, we adapted a Bayesian sequential prediction model [26], incorporating perceptually plausible constraints to the model’s resources. Fig 3 shows a schematic of the model and its outputs.

The input to the model is a sequence of observations {*x*_*t*_}; in our case, the observations are the pitches from the melody stimulus. The model sequentially builds a predictive distribution of the next observation at time *t* + 1 given the previous observations: *P*(*x*_*t*+1_|*x*_1:*t*_). Observations are assumed to be distributed according to some probability distribution with unknown parameters *θ*. At unknown changepoint times, the parameters *θ* change, and all following observations are drawn from this new distribution, independent of observations before the change. Observations between changepoints drawn from the same distribution form a *run*, and the time between changepoints is referred to as the *run-length*. If the most recent changepoint (or equivalently, the current run-length) were known, the independence of observations across changepoints could be used to simplify the prediction equation: given the current run-length *r*_*t*_, *P*(*x*_*t*+1_|*r*_*t*_, *x*_1:*t*_) = *P*(*x*_*t*+1_|*r*_*t*_, *x*_*t* − *r*_*t*_+1:*t*_).

Because changepoints must rather be *inferred* from the observations, the model maintains multiple hypotheses across all possible run-lengths and integrates them to predict the next observation:

$$\begin{array}{c} P\left(x_{t+1}|x_{1:t}\right)=\sum_{r_{t}}P\left(x_{t+1}|r_{t},x_{t-r_{t}+1:t}\right)P\left(r_{t}|x_{1:t}\right) \end{array}$$

In the sum, the prediction given run-length *r*_*t*_ (the first term) is weighted by the model belief that the current run-length is *r*_*t*_ (the second term). With each incoming observation, these run-length beliefs are incrementally updated and a new belief is added with length zero and weight *π*, the change-prior, re-weighting the predictions in the sum. The change-prior is a parameter of the model that represents the prior belief that a change will occur at any time before evidence for a change is observed (see S1 Text). Maintaining multiple run-length hypotheses is a key aspect of the model. Rather than making a hard decision about when a changepoint occurs and “resetting” the statistics, the model uses the observations as evidence to weight different interpretations of the sequence.

In the present application of the model, the generating distribution is assumed to be a *D*-dimensional multivariate Gaussian with unknown mean and covariance structure, where the dimensionality *D* specifies the amount of temporal dependence in the model. As new observations come in, the model incrementally collects sufficient statistics whose form depends on *D* (see Methods). Here, we ask whether human behavior from Experiments 1–2 can be captured by a model that collects marginal lower-order statistics (*D* = 1, i.e., mean and variance) or if higher-order statistics (*D* = 2, i.e., mean, variance, and covariance) are needed; we refer to these two versions of the model as the *LOS model* and *HOS model*, respectively.

#### Perceptual parameters

As described thus far, the model can maintain an infinite number of hypotheses, predicting the next observation in a Bayes-optimal manner [26]. To introduce more perceptual plausibility, we imposed two constraints on the model. First, a memory parameter (*m*) represents finite working memory capacity, limiting how many past observations can be used to build predictions and update run-length beliefs. Second, an observation noise parameter (*n*) sets a lower bound on prediction uncertainty by adding a constant variance to the predictive distributions (see Methods for details).

#### Model output

Fig 3b shows the outputs of the model for an example sequence of observations (top-foreground). The *predictive distribution* (top-background) integrates predictions across all hypotheses and provides a single posterior prediction given previous observations. After a new observation is “observed”, the *surprisal* (Fig 3b: middle) measures how well the observation was predicted by the model:

$$\begin{array}{c} S_{t}=-\text{log}\;P\left(x_{t}=X_{t}|x_{1:t-1}\right) \end{array}$$

where *S*_*t*_ is the surprisal for *X*_*t*_, the new observation at time *t*, and *P*(*x*_*t*_ = *X*_*t*_|*x*_1:*t*−1_) is the predictive probability of observing *X*_*t*_. Note surprisal is inversely related to the predictive probability—an observation with low probability has high surprisal, and vice versa.

We also derive a *change probability*—the probability a change has occured—from the run-length beliefs, *P*(*r*_*t*_|*x*_1:*t*_). The probability that a change has *not* occurred before time *t* is equal to the belief that the current run-length is equal to the length of the entire observed sequence (i.e., *P*(*r*_*t*_ = *t*|*x*_1:*t*_)); the probability that *at least* one change has occurred is then the converse of this, or the sum of beliefs in run-lengths less than the length of the observed sequence:

$$\begin{array}{c} P\left(\text{Change}|x_{1:t}\right)=1-P\left(r_{t}=t|x_{1:t}\right)=\sum_{r^{\prime }<t}P\left(r_{t}=r^{\prime }|x_{1:t}\right) \end{array}$$

An example of how this change probability unfolds is shown in Fig 3b (bottom). Importantly, the model is causal, so the predictive distribution, surprisal, and change probability only depend on the preceding observations and are updated sequentially *with each new observation*.

Finally, to collect responses from the model that are comparable to those collected from human listeners in Experiments 1–2, we use a simple decision rule. At the end of the melody (i.e., post-trial), the model makes a *change decision* by comparing the final change probability to a decision threshold:

$$\begin{array}{c} \text{Change}\;\text{decision}=\{\begin{array}{cc} \text{Yes}, & P(\text{Change}|x_{1:T})\geq \tau \\ \text{No}, & P(\text{Change}|x_{1:T})<\tau \end{array} \end{array}$$

where *T* is the full melody length and the threshold *τ* is an additional parameter of the model. We then define the model *changepoint* as the earliest time at which the change probability exceeds this threshold:

$$\begin{array}{c} \text{Model}\;\text{changepoint}=\underset{t}{\text{arg}\;\text{min}}\left\{P\left(\text{Change}|x_{1:t}\right)\geq \tau \right\} \end{array}$$

#### Perceptual parameters and model behavior

We first examined the model detection performance for different sets of model parameters: memory (*m*), observation noise (*n*), change-prior (*π*), and threshold (*τ*). Using a parameter sweep, we collected model change decision responses to the same stimuli used in Experiments 1–2 and measured model performance for each operating point in the sweep.

**Fig 4:**
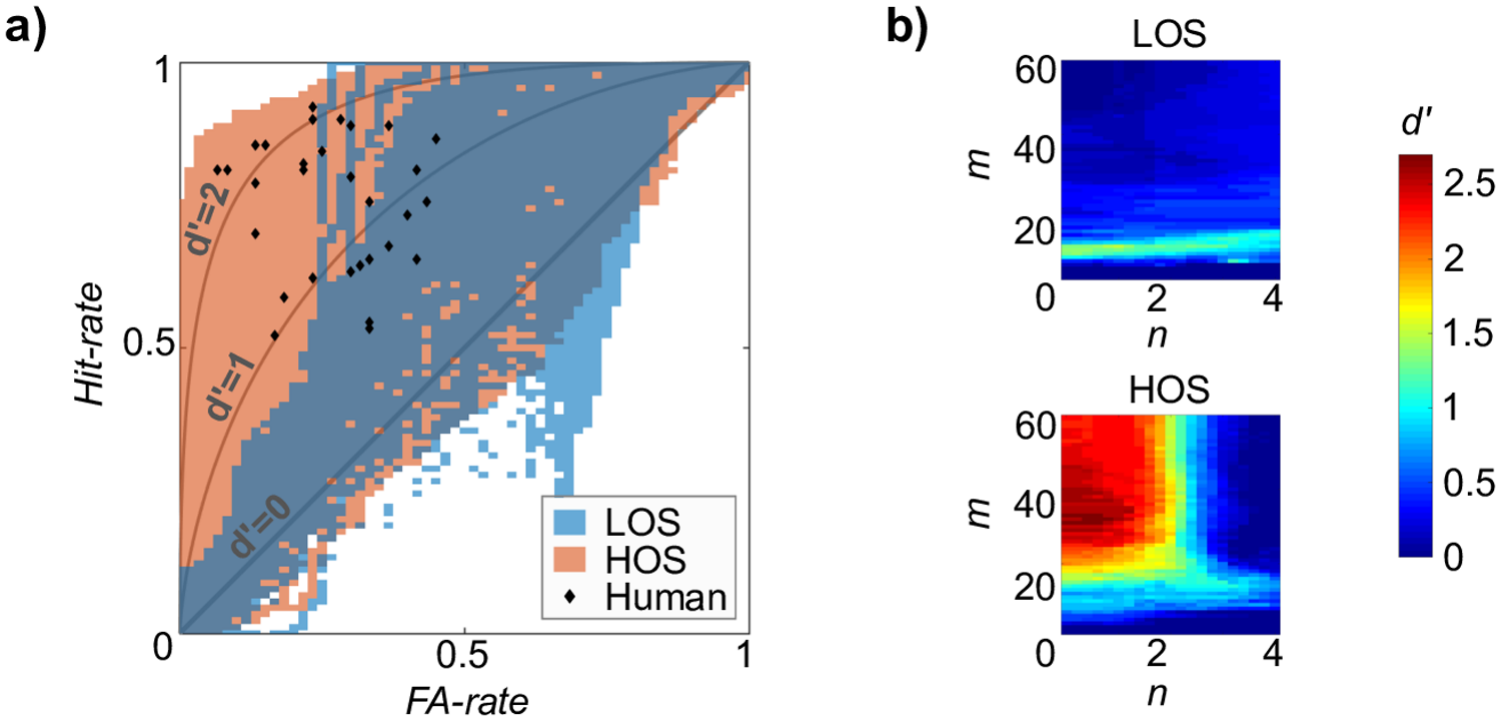
Range of model behavior in Experiment 1. Model detection performance measured at different operating points in a parameter sweep. a) Comparison of detection performance for LOS and HOS models displayed in ROC-space across the parameter sweep, with model type denoted by color. Each blue (red) coordinate indicates existence of a parameter set for the LOS (HOS) model yielding that performance. Individual human performance from Experiments 1 and 1b is overlaid, along with equal-*d*′ curves. b) *d*′ surface as a function of memory (*m*) and observation noise (*n*) parameters for LOS model (top) and HOS model (bottom). *π* and *τ* were held constant at 0.01 and 0.5, respectively.

Fig 4 shows model performance for Experiment 1. Performance is displayed in Receiver Operating Characteristic space (ROC-space); ROC-space is a method for visualizing the trade-off between Hit- and FA-rates in system performance at multiple operating points (i.e., parameter sets); the upper-left corner is perfect performance (Hit = 1, FA = 0), and the diagonal is chance performance (Hit = FA). Fig 4a displays the coverage of model performance in ROC-space for the LOS and HOS model (in blue and red, respectively); for example, at every red-colored coordinate in ROC-space, there is a set of parameters {*m*, *n*, *τ*, *π*} in the HOS model with that performance (i.e., Hit- and FA-rate). In this manner, we can compare the *range* of performance between the two models across the entire parameter sweep. Individual human performance from Experiments 1 and 1b (with the same stimuli, *N* = 31) and equal-*d*′ curves are overlaid in the same space for comparison. Results from Experiment 2 were similar.

There is a clear contrast in the range of performance in ROC-space between LOS and HOS models, with the HOS model having both wider coverage and higher ceiling performance overall compared to the LOS model. While the LOS model only overlaps with poorer performing subjects (*d*′ < 1.5), the HOS model overlaps with all human performance points. Additionally, human performance never exceeds the range of the HOS model, indicating that with unconstrained resources (i.e., infinite memory and zero observation noise) the HOS model can act as an “ideal observer”, providing an upper bound for human performance.

Fig 4b shows the *d*′ surface for the LOS model (top) and HOS model (bottom) as a function of the two perceptual parameters, allowing us to assess which parameters are responsible for the performance variability seen in Fig 4a for each model. With the LOS model, the memory *m* is largely responsible for performance variability, with only a narrow band around *m* = 10 where the LOS model performs well above chance (*d*′ = 0). The HOS model performance, on the other hand, varies jointly with both memory *m* and observation noise *n*, with the best performance around {*n* = 0, *m* = 30}.

#### Fitting the model to subject behavior

We fit the model parameters to each subject from Experiments 1–2. There was very high between-subject variability in performance (e.g., see human performance plotted in ROC-space in Fig 4a), so we examined how the parameters from the fitted model explain this variance. Model performance was measured for each set of parameters in the parameter sweep, and the best set of parameters was selected for each subject using minimum Euclidean distance between model and subject performance. Performance was measured using hit- and FA-rate within each change direction, which provided a more stringent criterion for distinguishing between parameters with equal overall hit- and FA-rates.

**Fig 5:**
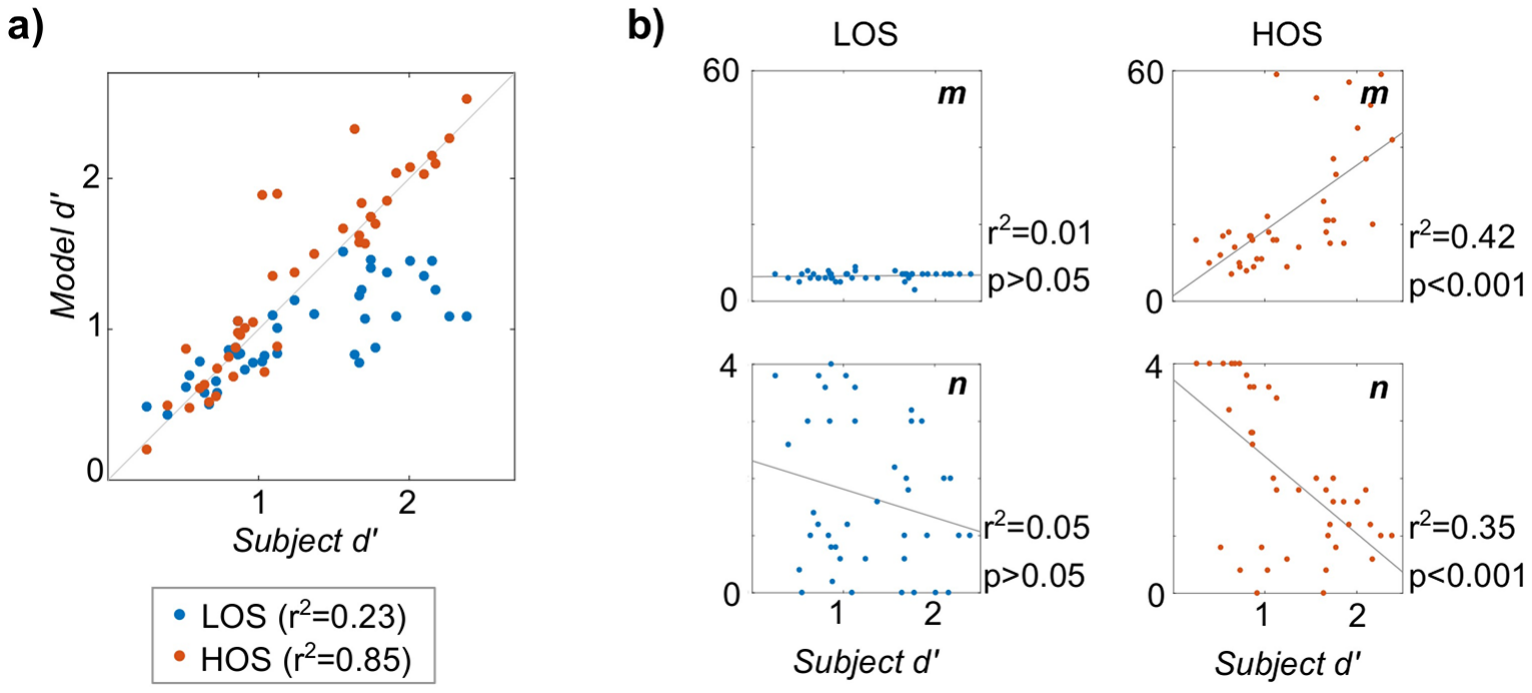
Model fit to subject behavior from Experiments 1–2. a) Subject *d*′ plotted against fitted model *d*′ for both LOS and HOS models, denoted by color. Legend shows *r*^2^-value from zero-intercept linear regression. b) Fitted perceptual parameters plotted against subject *d*′ for *m* (top) and *n* (bottom), with LOS model on the left and HOS model on the right. *r*^2^ and p-values shown for standard linear regression.

Fig 5 shows results from fitting the model to subjects from Experiments 1–2 (*N* = 41). In Fig 5a, subject *d*′ is plotted against model *d*′ for both LOS and HOS models. Using a linear regression with zero-intercept, the HOS model provided a better fit to subject behavior (*r*^2^ = 0.85, *p* < 0.0001) compared to the LOS model (*r*^2^ = 0.23, *p* < 0.0001), which cannot match the better-performing subjects.

Fig 5b shows the fitted perceptual parameters (*m* and *n*) plotted against subject *d*′ for the LOS and HOS models. With the LOS model (left), neither perceptual parameter has a significant linear relationship with subject *d*′ (*m*: *r*^2^ = 0.009, *F*(1, 39) = 0.359, *p* > 0.05; *n*: *r*^2^ = 0.05, *F*(1, 39) = 2.03, *p* > 0.05). With the HOS model (right), *both* memory and observation noise exhibit significant linear relationships with subject *d*′ (*m*: *r*^2^ = 0.423, *F*(1, 39) = 28.6, *p* < 0.0001; *n*: *r*^2^ = 0.352, *F*(1, 39) = 21.1, *p* < 0.0001), with higher memory and lower observation noise corresponding with better subject performance. Similar analysis with the other model parameters (*π* and *τ*) showed no correlation with subject *d*′ for either model.

To determine whether both perceptual parameters are needed to fit the HOS model to subject behavior, we tested a reduced model with only one of the perceptual parameters free. The memory-only HOS model, holding observation noise at *n* = 0, provided a poorer fit compared to the full HOS model shown in Fig 5a (*r*^2^ = 0.60, *p* < 0.001), as did the observation noise-only HOS model, holding memory at the maximum stimulus length *m* = 60 (*r*^2^ = −0.29, *p* < 0.001). Both memory and observation noise are needed as constraints to the model to fit the full range of human behavior.

Additionally, we compared the model changepoints to the RTs collected in Experiment 1b. Using a linear regression, the HOS model showed a significant linear relationship between model changepoint and subject RTs (*r*^2^ = 0.05, *F*(1, 1512) = 86.9, *p* < 0.0001), while the LOS model showed no significant relationship. Importantly, the model was fitted using the Yes/No response only and not the RTs themselves.

### Electroencephalography

Next, we examined neural underpinnings of higher-order stochastic regularities in the brain. In an experiment structured similarly to Experiments 1 and 2 above, listeners were asked to detect changes in stochastic melodies while EEG was simultaneously recorded from central and frontal locations on the scalp. Stimuli were generated at two levels of entropy (i.e., one change degree) with both INCR and DECR change direction.

#### Deviance response according to melody entropy

We first examined effects of melody entropy on ERPs to individual tones. Magnitude of frequency deviation (Δ*F*) is known to affect ERP morphology [32], so to determine any additional effect of entropy on the ERP, we computed average ERPs for both small and large Δ*F* (Δ*F* = 1 and 4 s.t. or semitones from the previous tone) at each entropy level (LOW and HIGH). Large Δ*F* tones are more rare in LOW entropy melodies compared to HIGH entropy melodies, so we might expect a deviance response that reflects this difference in relative occurrence (as seen in [32]). Δ*F* = 1 was chosen because it is the most frequent in both entropy levels, and Δ*F* = 4 was chosen to maximize frequency deviation magnitude while ensuring an adequate number of trials in the LOW entropy condition. We note that this analysis is more closely aligned with lower-order statistics, where deviance is always proportional to Δ*F*.

**Fig 6:**
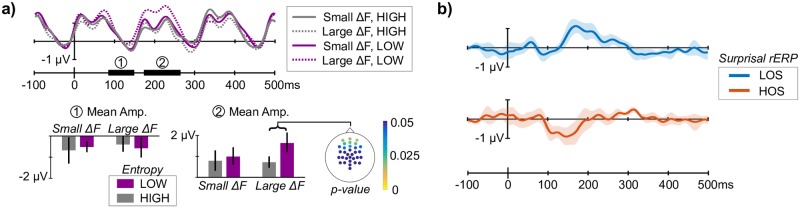
Contextual effects on tone ERP. a) Grand-average ERPs (top) for large and small Δ*F* in LOW and HIGH entropy melodies show a positivity for large Δ*F* in LOW entropy context around 200ms after tone onset. Mean amplitudes are shown for ① and ② time windows (bottom). Scalp map (right) shows frontal distribution of t-test *p*-values for large Δ*F* deflection between entropy contexts. b) Using model surprisal, regression-ERP analysis teases out distinct components depending on the set of statistics used in the model: a positivity 150-230ms after onset with LOS surprisal (similar to a) above) and a MMN-like negativity 100-200ms after onset with HOS surprisal. Error bars show 95% bootstrap confidence interval across subjects.

Fig 6a (top) shows grand-average ERPs for the four conditions averaged across frontal electrodes, which exhibited the strongest effect (described below). There is a divergence around 150-280 ms post-onset, where the ERP to large Δ*F* in LOW entropy (purple-dotted line) increases relative to the corresponding ERPs with the same Δ*F* (gray-dotted line) or the same entropy context (purple-solid line). Fig 6a (bottom) shows the mean amplitude in two time windows: ① 90–150ms and ② 170–260ms, corresponding roughly to N1/MMN and P2 time ranges [32]. A repeated-measures ANOVA with 2 factors (entropy and Δ*F*) applied to the later window showed a main effect of entropy (*F*(1, 7) = 7.49, *p* < 0.05) and a trend due to Δ*F* (*F*(1, 7) = 4.57, *p* < 0.07) with no interaction effect. Considering large-Δ*F* amplitudes only, a post-hoc paired t-test showed a significant difference between LOW and HIGH entropy contexts (*p* < 0.05). We performed the same t-test for each electrode; Fig 6a (bottom, far right) shows the *p*-values by electrode plotted on the scalp, with significant differences at frontal electrodes only. Similar analysis on the earlier window ① showed no effects of frequency deviation or entropy context.

An MMN response is notably absent from the ERPs in Fig 6a, even though large frequency deviations are rare in LOW entropy melodies. Assuming an MMN response in the brain to regularity deviations, this indicates a discrepancy between the “regularity” as defined in this analysis and the regularity collected by the brain: the MMN response is not well-differentiated by frequency deviation alone, and therefore it does not show up in this analysis. To see an MMN response, we need the proper definition of regularity in our analysis.

#### Deviance response according to model surprisal

The model outputs *surprisal* as a continuous measure of regularity violation, where the regularity is defined by the statistics collected by the model. We used a linear regression analysis to find contributors to the tone-elicited ERPs attributable to surprisal from the LOS and HOS models fit to individual subject behavior [33, 34]. The resulting regression ERPs (or rERPs) give a fitted regression to single-trial ERPs at each time-point for each measure of surprisal, and their interpretation is straightforward: the surprisal rERP shows the change in the baseline ERP for a unit increase in surprisal (see Methods).

Fig 6b shows the surprisal rERP for the LOS model (top) and HOS model (bottom). The rERPs show two distinct contributors to the ERP differing both in polarity and latency, with the LOS-rERP containing a positive deflection around 150–250ms post-onset and the HOS-rERP containing a negative deflection around 100–200ms.

To test the significance of these rERP deflections, we applied a linear mixed effects (LME) model to single trial amplitudes in the same two windows as the analysis above: 90-150ms and 170-260ms after tone onset, roughly corresponding to N1/MMN and P2 time windows. LME models are well-suited for testing single-trial effects with unbalanced designs [35], which is the case with surprisal (by definition, there are fewer surprising events than unsurprising events). In the later time window, the LME model showed a significant effect of LOS-surprisal (*p* < 0.01) on mean amplitude and no effect from HOS-surprisal. The same model applied to mean amplitude in the earlier time window showed the opposite: no significant effect from LOS-surprisal and a significant effect from HOS-surprisal (*p* < 0.001). This analysis shows deviance responses in the tone-ERP that differ depending on the statistics, or regularities, collected by the model, and an MMN-like response only to tones surprising according to the higher-order statistics of the preceding melody.

#### Disruption in phase-locking at model changepoint

**Fig 7:**
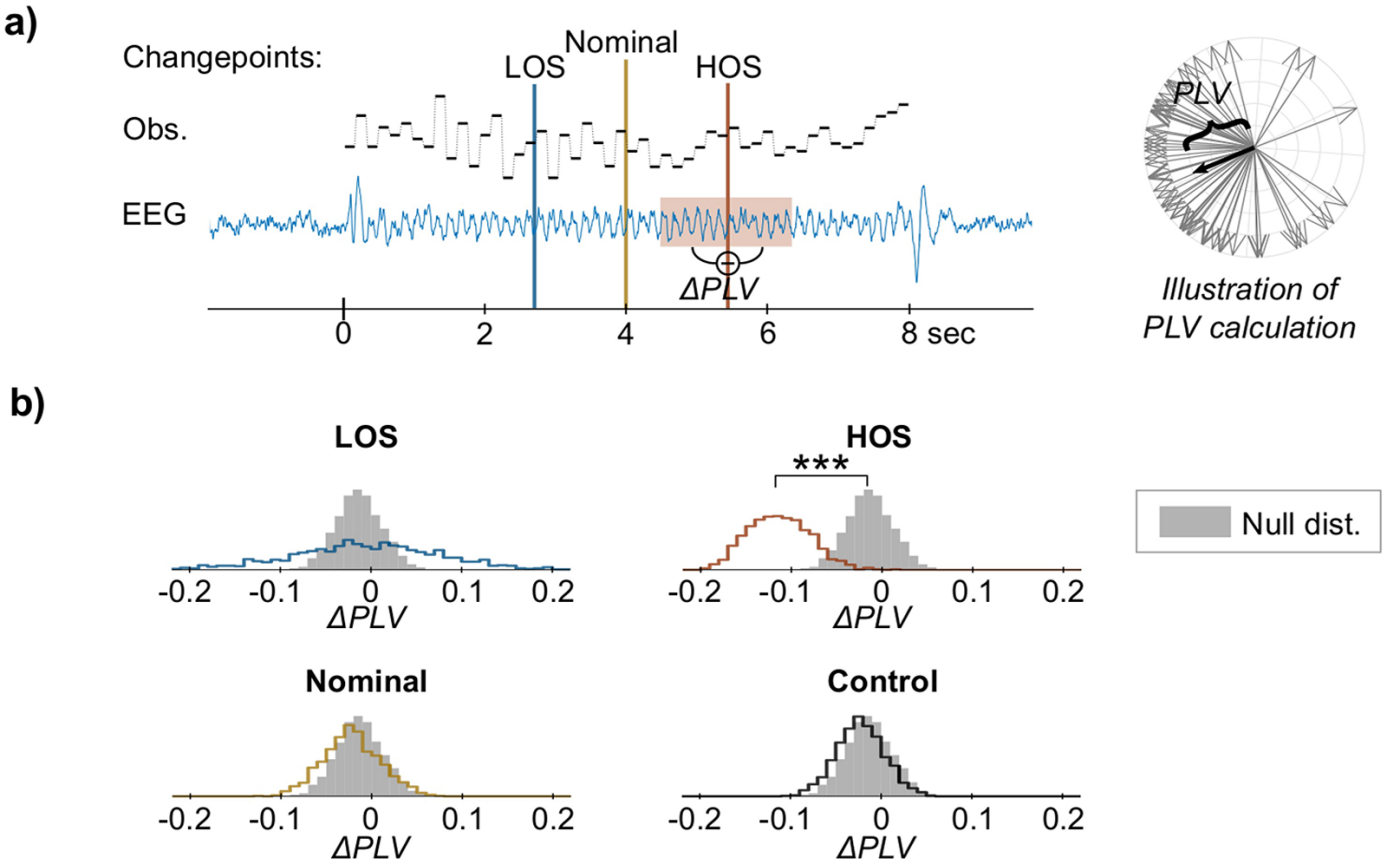
Phase-locking analysis at model changepoints. Δ*PLV* is used to measure disruptions in phase-locking of EEG to the tone presentation rate (6.25 Hz) at the time when the model detects a change in the stimulus (i.e., at the changepoint). a) Illustration of Δ*PLV* calculation. *PLV* measures phase agreement across trials independent of power; an example *PLV* calculation (right) shows the phase of individual EEG trials (in grey)—*PLV* is the magnitude of the mean of these normalized phasors (in black). Δ*PLV* is then the difference in *PLV* within a 7-tone (1-sec) window before and after the changepoint (left, shown at the HOS changepoint in the melody). For each subject, Δ*PLV* was calculated for three sets of changepoints: the changepoints output from the LOS and HOS models, and the nominal changepoint (i.e., midpoint) used to generate the stimuli. Additionally, as a control, the same HOS changepoints were applied to responses to no-change stimuli. b) Empirical distributions of Δ*PLV* at the LOS-, HOS-, Nominal-, and Control-changepoints (line) calculated by bootstrap sampling across subjects, along with the null distribution (solid gray) calculated by performing the same analysis with random sampling of the changepoint position. This null distribution estimates variability in Δ*PLV* present throughout the melody. Significant change from zero *and* from the null distribution is seen in the HOS-changepoint only.

We examined neural phase-locking to tone onsets before and after changepoints obtained from the LOS and HOS models. Phase-locking at the tone presentation rate (6.25 Hz) was measured from EEG data averaged across all 32 electrodes using the phase-locking value (*PLV*). *PLV* provides a measure of the phase agreement of the stimulus-locked response across trials, independent of power [36]. The difference in PLV before and after the changepoint (Δ*PLV*) measures the disruption in phase-locking at that time (see Fig 7a for illustration of Δ*PLV* calculation).

Δ*PLV* was measured at four sets of changepoints: the LOS and HOS model-changepoints, the nominal changepoint, and a control condition. The nominal changepoint (i.e., the midpoint) is the time where the generating distributions before and after have the greatest contrast. As a control for this analysis, HOS-changepoints were randomly assigned to control trials to ensure that any difference in PLV was due to the neural response recorded during change trials, and not simply due to the position of the changepoints.

Fig 7b shows the bootstrap distributions of the mean Δ*PLV* for each set of changepoints (lines). A paired t-test shows a significant decrease in *PLV* at the HOS-changepoints (*p* < 0.001), while there was no significant difference for the other changepoints. We also tested the Δ*PLV* measured at the changepoints against the variation in phase-locking present throughout the melody by estimating a null distribution, sampling null-changepoints at random positions in the melody and calculating Δ*PLV*. There was again a significant difference for the HOS-changepoints only (*p* < 0.001). These results together indicate there is a disruption in phase-locking that is specifically related to the changepoints obtained from the fitted HOS model.

## Discussion

How the brain extracts information from stochastic sound sources for auditory scene analysis is not well understood. We investigated stochastic regularity processing using change detection experiments, where listeners detected changes in the entropy of pitches in melodies. Results from Experiments 1–2 confirmed results from previous work showing that listeners represent information about stochastic sounds through statistical estimates [6, 8]. Listeners’ detection performance scaled with change degree (Experiments 1, 1b) and with the length of the sequence (Experiment 2), consistent with the use of a sufficient statistic to detect changes: a larger change in the statistic and a larger pool of sensory evidence both improved detection performance.

### What statistics are collected by the brain?

We introduced a perceptual model for stochastic regularity extraction and applied this model to the same change detection experiments as our human listeners. We used different sets of statistics in the model to determine which best replicate human behavior: a lower-order statistics (LOS) model that collects the marginal mean and variance of tone pitches or a higher-order statistics (HOS) model that additionally collects the covariance between successive tone pitches. Comparing the performance range for LOS and HOS models to human performance, we showed that higher-order statistics are necessary to capture all human behaviors, while lower-order statistics are insufficient to capture the full range of subject behaviors. This disparity strongly suggests the brain is collecting and using higher-order statistics about the temporal dependencies between incoming sounds. Furthermore, the model revealed effects in EEG that are only discernible using higher-order statistics: ERP evidence showed an MMN response elicited by tones that are surprising according to the higher-order statistics of the preceding melody, and cortical phase-locking was disrupted at the changepoints specified by the HOS model.

Interestingly, both LOS and HOS models were able to replicate behavior from poorer performing subjects (*d*′ < 1.5), but the LOS model is unable to mirror behaviors with high hit-rates without also increasing the FA-rate (Fig 4a). Intuition states that marginal statistics within the local context (i.e., short memory or small *m*) might be effective for detecting changes in local variance in the fractal sequences; this notion is supported by the model, where *m* = 10 tones yields the best LOS model performance (Fig 4b). Yet this local LOS model, with limited sampling in the statistics collected, is unable to match the performance exhibited by better performing subjects. In other words: if listeners (or the LOS model) rely solely on *marginal statistics*, then their ability to accurately flag changes in random fractal structure is highly constrained. Furthermore, relying on low-order statistics should elicit an effect of the direction of change (from low to high entropy or vice versa) on the hit-rates. Behavioral data shows no such effect of change direction on behavioral hit-rates (Experiments 1 and 1b), which further corroborates that listeners cannot be solely relying on lower-order statistics.

While these results strongly argue for the brain’s ability to track higher-order statistics in sound sequences, they do not disagree with previous work demonstrating sensitivity to lower-order statistics [8, 9]. Rather, by designing a task in which higher-order statistics are beneficial, we show that listeners are additionally sensitive to the temporal covariance structure of stochastic sequences. We also do not argue that the statistics collected by the brain are limited to these, but could include longer-range covariances. We performed the same analysis using a *D* = 3 model that collects covariance between non-adjacent sounds, but it did not provide any improvement over the *D* = 2 (HOS) model. This merely means that for our stimuli, there was no additional information to aid in change detection beyond the adjacent covariances. Additional experiments with stimuli that specifically control for this are needed to determine the extent of the temporal range of statistics collected by the brain.

### Individual differences revealed by stochastic processing

By their very nature, the stimuli used here exhibit a high degree of irregularity and randomness across individual instances of sequences. For the listener, deciding where the actual change in regularity occurs in a particular stimulus is a noisy process that arises with some level of uncertainty. Perceptually, most trials do not contain an obvious “aha moment” when change is detected; rather, the accumulation of evidence for statistical change emerges as a gradual process. Similarly from a data analysis point of view, determining the exact point of time when the statistical structure undergoes a notable change is a nontrivial problem, given that the perception of statistical change is not binary but continuous and varies both between trials *and* between listeners. As such, the study of stochastic processing hinges on the use of a model that is well-matched to the computations occurring in the brain, combining the right granularity of statistics with the right scheme for cue integration and decision making. And with the introduction of perceptual parameters to the model, we gain flexibility in the behaviors that can be reproduced by the model with clear interpretation as to the computational constraints leading to these behaviors.

Taking a close look at individual differences through the lens of the model, we were able to inspect underlying roots of this variability. Rather than simply a difference in decision threshold (i.e., “trigger-happiness”), we argue the variability across listeners was due to individual differences in the limitations of the perceptual system. We incorporated these limitations into the model via perceptual parameters. The memory parameter represents differences in working memory capacity [37, 38], and the observation noise parameter represents individual differences in pitch perception fidelity [39]. We should note that these parameters may also be capturing other factors that affect listener performance like task engagement, neural noise, or task understanding, which could be contributing noise to these results.

By fitting the model to individual listeners through their behavior, we showed correlates between human performance and the perceptual parameters of the model, and we found that neither perceptual parameter alone was adequate to fit all subjects. Rather than a nuisance, we see the inter-subject variability in these results as a consequence of individual differences in the perceptual system that are amplified by the uncertainty present in stochastic processing.

### Neural response depends on statistical context

We found effects of the statistical context on the neural response. First, examining ERP responses to individual tones, we found an enhanced P2 response to large frequency deviations in low-entropy melodies compared to high-entropy melodies and a frontal distribution of this difference consistent with sources in the auditory cortex. This result corresponds with previous work where large frequency deviations that were *less likely* given the previous context showed an enhanced P2 amplitude [32]. Similarly, we interpret this result reflecting a release from adaptation, where the low-entropy melody has a narrow local frequency range. Importantly, we do not see an MMN effect, arguably because frequency deviation alone is too crude to provide an adequate definition of “deviant” with our stochastic stimuli: large frequency deviations do not always violate the regularities in our stimuli, which may explain the lack of an observable MMN in the average differential response.

Using the fitted model, we were able to tease out distinct surprisal effects on the tone ERP that differ both in statistics and in temporal integration window: the LOS surprisal measured how well each tone was predicted by the lower-order statistics of the local context, while the HOS surprisal measured how well each tone was predicted by the higher-order statistics of the longer context, as fit by the model to individual behavior. Because LOS and HOS surprisal are partially (and unavoidably) correlated, both LOS and HOS surprisal were included in a single regression in order to find components in the ERP that correlate with each *independent of the other* [34].

We found an enhanced P2 amplitude with increasing LOS surprisal that is similar in amplitude and latency to the P2 difference discussed above; indeed, LOS surprisal provides a similar definition of regularity to the ERP analysis based on melody entropy above, for large frequency deviations are always “deviants” according to the lower-order statistics. We again attribute this increased P2 to a release from adaptation. Consequently, we can then attribute the MMN response to HOS surprisal as a deviance response according to higher-order statistics *independent from* lower-order adaptation effects.

There has been much discussion on whether the MMN response is truly a deviance response or merely due to adaptation [40, 41]. Many experiments suffer from confounding frequency deviance with regularity deviance, making it difficult to definitively attribute MMN to one or the other. With our stochastic stimuli differing in higher-order statistics, we were able to disentangle the two interpretations. We again stress that this result is not in conflict with previous results showing effects of lower-order statistics on the MMN [8, 42], because deviants in these studies could also be considered deviants according to their higher-order statistics (i.e., the HOS model reduces to the LOS model when the covariance between sounds is zero).

Finally, we found a disruption in the brain’s phase-locked response to tone onsets that coincides with HOS model changepoints, where the model detects a change in the higher-order statistics of each stimulus. Contrasting various controls using different estimates of when the change point occurs, we observed a notable phase disruption with changes in higher-order statistics only. The change in phase synchrony across trials could be due to the combined modulation of multiple ERPs to tones following the changepoint, or it could reflect a change in the oscillatory activity of the brain, which has been shown to correspond with both changes in predictive processing and attentional effects [43, 44]. Further experimentation is needed to determine the source of this disruption. Importantly, this analysis takes into account the stochastic nature of the stimuli by interpreting the statistical structure of each stimulus through the model, rather than with the changepoint used to generate the stimuli (i.e., the “nominal” changepoint).

### A model for Bayesian predictive processing in the brain

The model presented here fits in nicely with existing theoretical formulations for predictive processing and object formation in perception [2, 45], as well as Bayesian descriptions of the perceiving brain [46–48]. Importantly, the model does not assume stationarity in the sound environment, and it can adapt to changes in regularity at any time. To achieve this, the model hypothesizes two mechanisms in the brain: first, the brain builds representations by collecting statistical estimates from sounds over time; second, the brain maintains multiple hypotheses for how to interpret the previously heard sound sequence. These hypotheses are represented explicitly in the model by statistical estimates collected over different time-windows, each of which gives a prediction for future sounds. Prediction errors are then used to update the beliefs in each hypothesis, weighting hypotheses proportional to the amount of evidence relative to alternative hypotheses. This competition between concurrent hypotheses is crucial for robust interpretation in the presence of uncertainty. As new sounds deviate from the prediction of the current best hypothesis, beliefs shift to a new dominant hypothesis (and set of statistical estimates) that better explains the previous sounds; the beliefs therefore reflect the dynamics of a changing environment.

While in this work we used the model to investigate processing of regularities in pitch, we believe the same machinery can be applied to other auditory dimensions (e.g., loudness, timbre, spatial location) and extended to other sufficient statistics to test different representations of regularities in sound. Additionally, the model is not limited to detecting changes, as was demonstrated here using a simple decision rule. Rather, it is a perceptual model of stochastic predictive processing that can operate in the presence of changes, as the brain does while perceiving real-world, dynamic sound environments.

## Methods

### Experiment 1

#### Stimuli

Stimuli were pure-tone melodies with tone frequencies determined by random fractals. Random fractals are stochastic processes with spectrum inversely proportional to frequency and with spectral slope *β* (1/*f*^*β*^). *β* parameterizes the entropy of the random fractal: as *β* decreases entropy increases, with *β* = 0 yielding a white-noise spectrum and the highest entropy. Four levels of entropy were used to create the stimuli, corresponding to *β* = 0, 1.5, 2, 2.5. Random fractals were generated by repeatedly applying the inverse Fourier transform to the 1/*f*^*β*^ spectrum with random phase, yielding many unique instances. These random fractals were standardized to remove any differences in mean and variance, then quantized and mapped to 35 frequencies in a quasi-semitone scale (15 frequencies/octave) centered on 330 Hz (range: 150–724 Hz). Melodies were synthesized using pure tones with 150ms duration and 10ms ramped onset and offset (squared cosine). Inter-onset interval between tones was 175ms.

All melody stimuli in Experiment 1 had a length of 60 tones. Stimuli with changes in entropy (“change trials”) were composed of two equal-length melodies with different entropy, one with the highest entropy (*β* = 0) and one with a lower entropy, resulting in three degrees of change (Δ*β* = 1.5, 2, 2.5). Both increasing- and decreasing-entropy trials (referred to as INCR and DECR, respectively) were included, resulting in six change conditions, as well as control trials with constant entropy at each entropy levels. There were 150 trials in total, with 15 trials per condition.

#### Participants

Ten participants (9 Female) were recruited from an undergraduate population (mean age: 18.7 years). All participants reported no history of hearing loss or neurological problems. Participants gave informed consent prior to the experiment and were paid for their participation. All procedures were approved by the Johns Hopkins Institutional Review Board (IRB).

#### Procedure

Stimuli were presented in randomized order in 3 blocks with self-paced breaks between blocks. During each trial, listeners were instructed to listen for a change in the melody; after the melody finished, participants responded via keyboard whether or not they heard a change. Feedback was given after each response in order to guard against task misunderstanding and ensure listeners had as much information as possible to perform the task well.

Listeners were not given explicit instructions about what they were listening for, but rather learned the task implicitly over the course of a training block prior to testing. Incorrect responses in the training block caused the same stimulus to be replayed with feedback (including an indication of when the change occurs, in the case of missed detections). Participants advanced to testing after completing at least 15 trials and correctly answering 5 consecutive trials (all participants completed training in under 30 trials).

Stimuli were synthesized offline as 16-bit, 44.1 kHz wav-files and presented via over-ear headphones (Sennheiser HD 595) at a comfortable listening level using PsychToolbox (psychtoolbox.org) and custom scripts in MATLAB (The Mathworks). Participants were seated in an anechoic booth in front of the presentation computer. The experiment duration was approximately 50 minutes.

### Experiment 1b

#### Stimuli

Stimuli were the same as in Experiment 1.

#### Participants

21 participants (14 Female) were recruited from an undergraduate population (mean age: 20.1 years). All participants reported no history of hearing loss or neurological problems. Participants gave informed consent prior to the experiment and were paid for their participation. All procedures were approved by the Johns Hopkins IRB.

#### Procedure

This experiment had the same procedure as Experiment 1, with the exception of how responses were collected. In this experiment, listeners responded in the middle of the melody trial as soon as a change was heard by pressing the space-bar. If the space-bar was not pressed before the end of the melody presentation, this was recorded as a negative response. Responses before the nominal changepoint of change trials (i.e., the midpoint) were considered false-alarms.

### Experiment 2

#### Stimuli

Stimuli in this experiment were similar to those in Experiment 1 with an additional manipulation of melody length. Along with the same change degree and direction conditions, there were three length conditions (20, 40, and 60 tones) with the change always occuring in the midpoint of the melody. For each of the 18 change conditions (3 Δ*β* x 2 direction x 3 length) and each of the 12 control conditions (4 *β* x 3 length), there were 8 trials, for a total of 240 trials.

#### Participants

Ten participants (6 Female) were recruited from an undergraduate population (mean age: 18.7 years). All participants reported no history of hearing loss or neurological problems. Participants gave informed consent prior to the experiment and were paid for their participation. All procedures were approved by the Johns Hopkins University IRB.

#### Procedure

Procedure was the same as in Experiment 1, including training and testing phases.

### EEG experiment

#### Stimuli

In this experiment, stimuli were based on an alternative parameterization of entropy using first-order Markov chains, which provided greater control over the distributions used to generate the melodies. Specifically, this allowed us to exclude tone repetitions from the melody stimuli to prevent any correlates in EEG due simply to repetition. Because none of the analyses or results are predicated on properties exclusive to random fractals, and both types of stochastic stimuli are perceptually similar, we treat both stimuli identically.

Melody stimuli were composed of 50 pure-tones with pitches sampled from 11 frequencies on a semitone scale (range: 247–440 Hz). For each melody, the first tone frequency was sampled uniformly from all 11 frequencies. Subsequent tone frequencies were drawn from a probability distribution based on a modified logistic curve centered on the previous observation with entropy parameterized by the logistic slope *k*,

$$\begin{array}{c} P_{k}\left(x_{t}|x_{t-1}\right)=\{\begin{array}{cc} 0, & x_{t}=x_{t-1}\\ \frac{A}{1+e^{-k|x_{t}-x_{t-1}|}}, & \text{otherwise} \end{array} \end{array}$$

where *x*_*t*_ and *x*_*t*−1_ are the current and former tone frequencies (in semitones) and *A* is a normalization constant. As *k* increases, this distribution becomes more biased towards smaller frequency steps and lower entropy, and it has maximum entropy at *k* = 0, a uniform distribution across the 10 frequencies (excluding the previous frequency). High-entropy sequences and low-entropy sequences were generated with *k* = 0 and *k* = 0.7, respectively. For change trials, *k* transitioned smoothly between the two extremes in the middle 10 tones of the melody (tones 21–30) to avoid obvious outliers from an abrupt change in the distribution.

There were 150 melody trials in this experiment: 50 trials for each change direction (INCR and DECR), and 25 control trials per entropy level (LOW and HIGH). Tones were 125 ms in duration and presented with inter-onset interval of 160 ms.

#### Participants

14 participants were recruited for this experiment, however six were excluded from EEG analysis because they had behavioral performance near chance (*d*′ < 0.5). Out of the remaining eight subjects, six were female, and the mean age was 20 years.

#### Procedure

The procedure in this experiment was similar to that in Experiments 1 and 2. Subjects were seated in an anechoic chamber with stimuli presented via in-ear earphones (Etymotic ER-2) at a comfortable listening level. Before each melody trial, a cross appeared in the center of the screen, and subjects were instructed to fixate on the cross to reduce eye movement artifacts. After the trial, subjects responded whether or not they heard a change via a response box.

#### Data recording and pre-processing

EEG was recorded using a BioSemi ActiveTwo system (Biosemi) with 32 electrodes placed in central and frontal locations on the scalp selected to maximize signal-to-noise ratio for neural signals originating in auditory centers of the brain [49, 50]. Six additional electrodes were placed on left and right mastoids, the nose, and alongside the eyes for re-referencing and blink artifact removal. Data was recorded at a sampling rate of 4096 Hz.

For each subject, EEG data were preprocessed with custom scripts in MATLAB using the FieldTrip toolbox (www.fieldtriptoolbox.org) and NoiseTools [51]. Continuous EEG was re-referenced to the left mastoid, filtered to 1–100 Hz (two-pass Butterworth, 3^rd^-order for high-pass and 6^th^-order for low-pass), and re-sampled to 256 Hz. The data was then cleaned in two stages using Independent Component Analysis (ICA) and Denoising Source Separation (DSS). First, continuous EEG data was epoched to 1 second segments; segments with amplitude range exceeding 3 s.d. from the mean by channel were excluded before applying ICA to identify components attributable to eye motion artifacts. These artifact components were removed from the continuous EEG data, and the ICA-cleaned data was epoched to melody trials. DSS was then used to enhance stimulus-locked activity; the top 5 DSS components that were most repeatable across melody trials were kept and projected back to sensor space, thus removing EEG signal not related to auditory stimulation [51].

#### Data analysis

We used regression to investigate effects of model surprisal on ERP responses based on the framework described in [33, 34]. For each subject, EEG data was further low-pass filtered at 30Hz (6^th^-order Butterworth) and epoched by tone with the 50-ms window preceding tone onset used for baseline subtraction. Outlier tone trials with amplitude exceeding 3 s.d. from the mean were excluded from the analysis.

We fit the following regression model to single-trial ERPs:

$$\begin{array}{c} y_{i}\left(t\right)=\beta_{0}\left(t\right)+S_{L}\beta_{L}\left(t\right)+S_{H}\beta_{H}\left(t\right)+\epsilon_{i}\left(t\right) \end{array}$$

where surprisal from the LOS model (*S*_*L*_) and the HOS model (*S*_*H*_) serve as predictors in the regression for the *i*^*th*^ single-trial ERP (*y*_*i*_). The regression contains an intercept term *β*_0_, which captures the baseline ERP response, and slope terms *β*_*L*_ and *β*_*H*_, which capture the differential response due to a unit change in *S*_*L*_ and *S*_*H*_, respectively. Finally, *ϵ*_*i*_ is the residual error for the i-th trial. Note that these terms are indexed by time, so the regression finds the linear relationship between regressors (*S*_*L*_ and *S*_*H*_) and the single-trial ERPs at each time point, yielding a regression-ERP, or rERP [34]. The regression was applied separately for each subject to EEG data averaged across all 32 electrodes.

We used phase-locking value (*PLV*) to measure neural phase-locking to tones. *PLV* is a measure of phase agreement across trials independent of signal power:

$$\begin{array}{c} PLV=\frac{1}{n}|\sum_{i=1}^{n}\phi_{i}/|\phi_{i}|| \end{array}$$

where the *ϕ*_*i*_’s are complex phasors extracted from the Fourier transform at the frequency of interest (6.25Hz, the tone presentation rate) for the *i*^*th*^ trial, and *n* is the number of trials. PLV was calculated separately for 1120ms (7-tone) epochs before and after the changepoints, and the difference, Δ*PLV* = *PLV*_*after*_ − *PLV*_*before*_, was used to measure the change in phase-locking at the changepoints. Only change trials correctly detected by both listener and model were included in this analysis.

For statistical testing, Δ*PLV* was compared to 0 (t-test) and to a null distribution (random permutation test) estimated by calculating Δ*PLV* from randomly sampled changepoints across the melody. The null distribution ensures any observed change in *PLV* at the changepoints is not simply due to the random variability in phase-locking present across the melody trial.

### Model

The perceptual model is an extension of the Bayesian Online Changepoint Detection model described in [26], which was designed to predict incoming observations sequentially given previous observations in the presence of unknown changepoints. Model code is available at https://engineering.jhu.edu/lcap/.

Because the model assumes observations are generated from a *D*-dimensional Gaussian distribution, there exists a closed-form solution for the predictive distribution that depends only on the sufficient statistics ${\hat{\theta }}_{t}^{\left(r_{t}\right)}=\left\{{\hat{\mu }}_{t}^{\left(r_{t}\right)},{\hat{\Sigma }}_{t}^{\left(r_{t}\right)}\right\}$, i.e., the *D*-dimensional sample mean and sample covariance at time *t* collected over the previous *r*_*t*_ observations [52]. We modify these sufficient statistics with perceptual parameters to add perceptually plausible constraints to the model. Observation noise (*n*) adds a constant variance to the predictive distribution, and the memory parameter (*m*) puts a limit on the number of past observations used in the prediction, effectively “forgetting” observations outside of this window. We then have a modified expression for the sufficient statistics for run-length *r*_*t*_ at time *t* that incorporates the two perceptual parameters:

$$\begin{array}{c} {\hat{\theta }}_{t}^{\left(r_{t}\right)}=\{\begin{array}{cc} \{{\hat{\mu }}_{t}^{\left(r_{t}\right)},{\tilde{\Sigma }}_{t}^{\left(r_{t}\right)}\}, & r_{t}<m\\ \{{\hat{\mu }}_{t}^{\left(m\right)},{\tilde{\Sigma }}_{t}^{\left(m\right)}\}, & r_{t}\geq m \end{array} \end{array}$$

where ${\tilde{\Sigma }}_{t}^{\left(r_{t}\right)}={\hat{\Sigma }}_{t}^{\left(r_{t}\right)}+n^{2}I_{D}$ is the sample covariance with added observation noise *n*, and *I*_*D*_ is the *D*-dimensional identity matrix.

## Supporting information

S1 Audio Example melody with high entropy, (*β* = 0). (WAV)

S2 Audio Example melody with medium-high entropy, (*β* = 1.5). (WAV)

S3 Audio Example melody with medium-low entropy (*β* = 2). (WAV)

S4 Audio Example melody with low entropy (*β* = 2.5). (WAV)

S5 Audio Example INCR stimulus with increasing entropy (*β* = 2 → 0). (WAV)

S6 Audio Example DECR stimulus with decreasing entropy (*β* = 0 → 2). (WAV)

S1 Fig Hit and False Alarm (FA) rates for Experiment 1. The difference in performance across change direction (INCR, DECR) as measured by *d*′ is due to increased FAs with increasing entropy. There was no effect of direction on hit rates alone.
(TIFF)

S2 Fig Hit and False Alarm (FA) rates for Experiment 2. Hit rates show a strong effect of both change degree and melody length, while FAs only show an effect of entropy.
(TIFF)

S1 Text Model update equations. Brief description of update equations for recursive estimation of run-length beliefs, with full treatment appearing in [26].
(PDF)
