# Supplementary figures and images for: Detecting change in stochastic sound sequences
Source: PLoS Comput Biol. 2018 May 29;14(5):e1006162. doi: 10.1371/journal.pcbi.1006162 (PMC5993325; doi:10.1371/journal.pcbi.1006162)

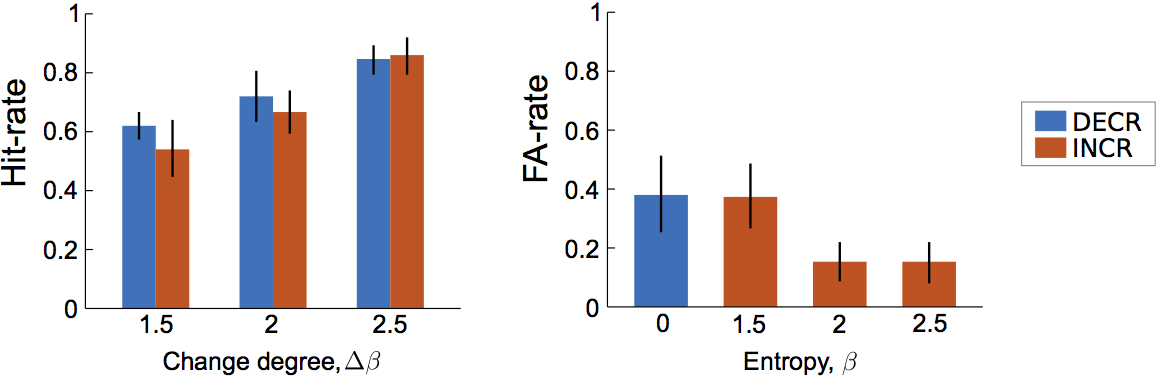

Supplement: S1 Fig — The difference in performance across change direction (INCR, DECR) as measured by d′ is due to increased FAs with increasing entropy. There was no effect of direction on hit rates alone. (TIFF) [file pcbi.1006162.s007.tiff]

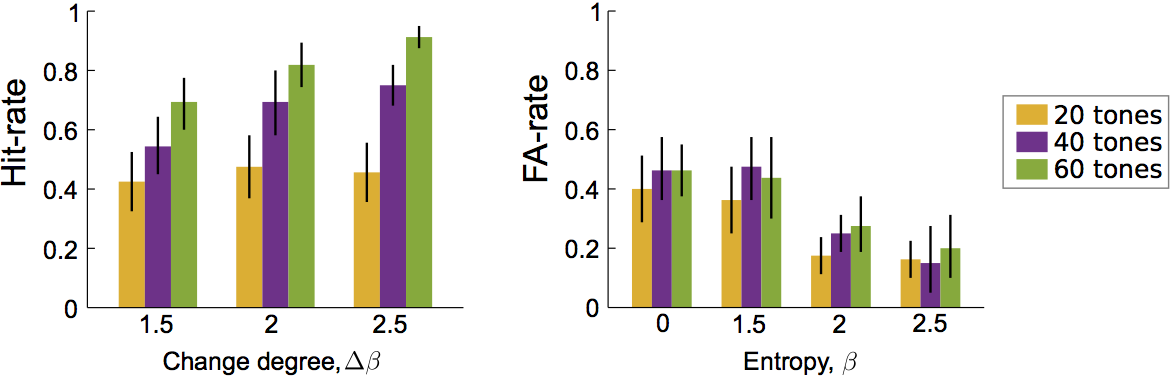

Supplement: S2 Fig — Hit rates show a strong effect of both change degree and melody length, while FAs only show an effect of entropy. (TIFF) [file pcbi.1006162.s008.tiff]
