# Supplementary material for: Detecting change in stochastic sound sequences
Source: PLoS Comput Biol. 2018 May 29;14(5):e1006162. doi: 10.1371/journal.pcbi.1006162 (PMC5993325; doi:10.1371/journal.pcbi.1006162)
Supplement: S1 Text — Brief description of update equations for recursive estimation of run-length beliefs, with full treatment appearing in [26]. (PDF) [file pcbi.1006162.s009.pdf]

S1 Appendix

Model Update Equations

After the observation has been observed, the model uses the predictive probability of the new observation under each run-hypothesis to update the run-length beliefs. The run-length beliefs can be expressed using Bayes rule as:

$$P(r_{t+1}|x_{1:t+1}) = \frac{P(r_{t+1}, x_{1:t+1})}{P(x_{1:t+1})}$$

where in the denominator $P(x_{1:t+1}) = \sum_{r_{t+1}} P(r_{t+1}, x_{1:t+1})$. We can then get a recursive update equation for $P(r_{t+1}, x_{1:t+1})$ in terms of $P(r_t, x_{1:t})$ by expressing it as a marginal probability and simplifying:

$$\begin{aligned} P(r_{t+1}, x_{1:t+1}) &= \sum_{r_t} P(r_{t+1}, r_t, x_{1:t+1}) \\ &= \sum_{r_t} P(r_{t+1}, x_{t+1}|r_t, x_{1:t}) P(r_t, x_{1:t}) \\ &= \sum_{r_t} P(r_{t+1}|r_t) P(x_{t+1}|r_t, x_{t-r_t+1:t}) P(r_t, x_{1:t}) \end{aligned}$$

The algorithm becomes tractable because the run-lengths can only grow incrementally by one for each new observation, so the first term in the sum, $P(r_{t+1}|r_t)$, is only non-zero for two values of r_{t+1}:

$$P(r_{t+1}|r_t) = \begin{cases} \pi, & r_{t+1} = 0 \\ 1-\pi, & r_{t+1} = r_t + 1 \\ 0, & \text{otherwise} \end{cases}$$

where π is the change-prior, the prior probability a changepoint has occurred after the most recent observation and the run-length drops to zero. π is generally small, siphoning off a sliver of probability mass from each run-length belief to allow for the possibility of a changepoint at any time.

Returning back to the summation above, each run-length belief is updated based on: 1) how well it predicts the new observation, and 2) its belief at the previous time. The conditioning on $P(x_{1:t+1})$ then yields a probability distribution over the possible run-lengths, where run-lengths that provide a better prediction of the observed sequence have a higher probability. These updated run-length beliefs are used to weight the predictions for the following observation, and so on.

The model is initialized at the beginning of each melody trial with the first changepoint known *a priori* before the beginning of the melody, $P(r_0 = 0) = 1$. The zero-length run-length hypothesis at each time (i.e., $r_t = 0$) is initialized with prior sufficient statistics collected over the entire stimulus set for each experiment.
